# Design and Control of a Trapezoidal Piezoelectric Bimorph Actuator for Optical Fiber Alignment

**DOI:** 10.3390/ma16175811

**Published:** 2023-08-24

**Authors:** Xinjie Wang, Jianhui Li, Xingfan Lu

**Affiliations:** School of Mechanical Engineering, Nanjing University of Science and Technology, Nanjing 210094, China; jh_li@njust.edu.cn (J.L.); l1728782484@163.com (X.L.)

**Keywords:** piezoelectric actuator, optical fiber alignment, structural design, control, simulation analysis

## Abstract

To align a pair of optical fibers, it is required that the micro actuators used be small and have the characteristics of high accuracy and fast response time. A trapezoidal piezoelectric bimorph actuator was proposed for pushing and pulling an optical fiber. Based on a mathematical model and finite element model established in this paper, we analyzed the output displacement and output force of the proposed trapezoidal piezoelectric actuator under the influence of structural parameters. Since the piezoelectric bimorph actuator had a hysteresis effect, we applied particle swarm optimization to establish a Prandtl–Ishlinskii (PI) model for actuator and parameter identification. Then, two control methods, namely feedforward control considering hysteresis effects and fuzzy proportional-integral-derivative (PID) control employing feedback, were proposed. Finally, a composite control model combining the two control methods with fewer tracking errors was designed. The results show that the output displacement of this actuator is larger than that of a rectangular one. Additionally, the fuzzy PID control has a lower response time (15 ms) and an overshoot (5%).

## 1. Introduction

Optical fibers are widely used for transmitting signals. One kind of light microswitch has been reported based on controlling interruption of light roads. Micro actuators are important to these switches and used for pushing and pulling fibers. However, with traditional actuators, it is hard to achieve the high accuracy and small volume required for the optical fiber alignment system. It is necessary to design and study specialized micro actuators and provide precise solutions for aligning optical fibers.

Many of the existing micro actuators have been driven mainly by electric heating elements [1], electrostatic plates [2], electromagnetic elements [3], piezoelectric components [4] and shape memory [5]. A direct-coupled optical switch [6] based on an electrothermal-driven cantilever was designed to have output displacement of 130 μm with a driven voltage of 44 V. As the temperature effect cannot be ignored, Dai et al. [7] coupled a mass-spring system with the actuator, while the method caused a size increase. Leung and his team [8] described a bimorph actuator for fiber alignment whose linear actuation is 60 μm and applied voltage is 100 V. Additionally, an electromagnetic optical switch [9] and an electro-thermal actuator for the switch [10] were discussed and proved to have practical value.

Compared with other types of micro actuators, piezoelectric actuators could be widely used because of their benefits in miniaturization and rapid response improvement, in fields such as medicine delivery [11], micro-robotics [12], and ultra-precision machining [13]. A stick-slip piezoelectric actuator [14] was optimized by using a flexible foot, allowing the actuator to work stably. To reduce response time, Rasid et al. [15] proposed a piezoelectric multi-layer actuator instead of electrostatic actuators to drive a micro-lens for imaging applications. Sumit et al. [16] studied the static displacement of a piezo actuator made of PZT-5H under high input voltage, and the result is helpful for designers to control the shape and vibration of bending piezo actuators. Though piezoelectric ceramics usually have strong hysteresis effects and require high driving voltage, the geometry of the piezoelectric element has a beneficial impact on the actuation. Many micro machines [17,18,19] use trapezoidal piezoelectric chips for higher energy density, force, bearing capacity, etc., which means it is feasible to change the shape of the element for improved output.

However, a piezoelectric actuator has the disadvantage of a strong hysteresis effect, and its small output displacement and output force still need to be improved. For practical application, it is necessary to consider how to reduce the impact of these defects when the actuator is in operation. Zorić et al. [20] proposed a method of controlling actuators based on fuzzy PID with particle swarm optimization. Chen et al. [21] combined the PI model and correction network to describe the hysteresis. Further, the Bouc–Wen model was used to describe the hysteresis effect, and the parameters of the model were identified by a particle swarm optimization method. Napole et al. [22] focused on fuzzy logic in combination with neural networks to design a feedback–feedforward algorithm.

In this paper, we compared the differences in output displacement and output force between trapezoidal and rectangular piezoelectrical bimorphs before selecting the geometry with better output characteristics. Then, a mathematical model and finite element model of the trapezoidal bimorph were developed and analyzed. Based on the analysis and available materials, we adjusted the structural parameters of the trapezoidal bimorph for subsequent experiments. After the hysteresis loop of the piezo actuator was measured, a particle swarm algorithm was employed to identify the parameters of the PI hysteresis model. Finally, we investigated feedforward control and fuzzy PID and sought to combine them for reduced tracking error.

## 2. Structure Design

### 2.1. Principle of Aligning Two Optical Fibers

**Figure 1:**
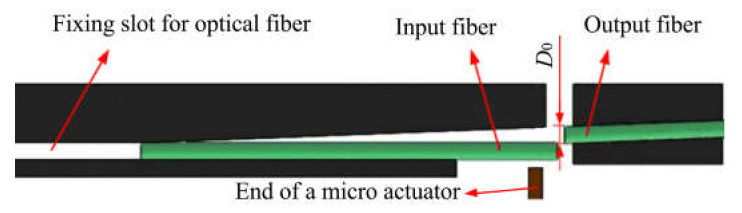
The working principle of the optical fiber alignment system.

For the alignment of a pair of optical fibers, the output fiber is completely fixed, and the input fiber can be regarded as a cantilever beam. They are actuated from an out-of-line to an in-line position and vice versa. If there is no input voltage, the end of the input fiber is in a free state without any pushing force. Therefore, the two fibers are staggered, and the optical path from the input fiber core to the output fiber core is disconnected. However, once the voltage is input, the piezoelectric actuator bends and deforms, and the two fibers are embedded in the slot. Thus, the ends of the two fibers will be aligned closely and the light path will be turned on. Figure 1 shows how a fiber alignment system is implemented with a micro actuator.

Different support modes will influence the operation of the actuator differently. There are mainly four support modes, namely simple support, rigid support, nodal support and cantilever support. We selected the cantilever support because the displacement of the actuator using this support is larger than that of actuators using the other modes.

The structure of a piezoelectric bimorph generally consists of five layers with three different materials. Two piezoelectric patches are attached to the upper and lower surfaces of the middle metal layer using an adhesive. In addition, the polarization direction of the piezoelectric layers is usually along the thickness direction. Considering the polarization direction and the conductivity of the bonding layer, we decided to use a parallel electrical connection to drive and control the actuator.

Additionally, the middle layer was made of beryllium bronze because it has good mechanical and physical properties [23]. For the material of the piezoelectric layer, Pb(Zr,Ti)O_3_-based ceramics were the first choice due to their good piezoelectric properties and low cost. And, they are stable under normal temperature conditions [24]. For example, the Curie temperature of PZT-5H is at 190 °C, and Yang’s modulus of poled PZT-5H is 83 GPa at room temperature [25], which was suitable for the actuator.

### 2.2. Structure of Piezo Actuator for Optical Fiber Alignment

**Figure 2:**
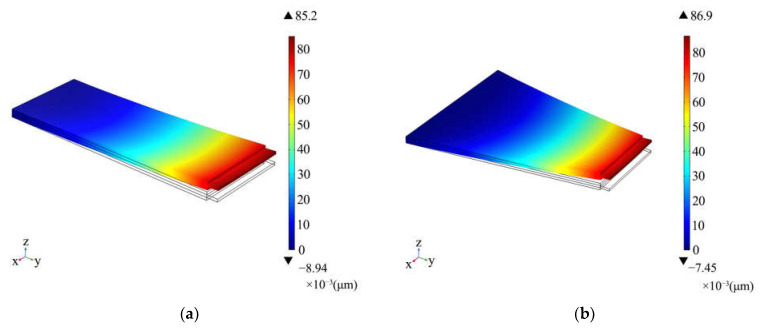
Finite element simulations of deformation of (**a**) the rectangular piezoelectric bimorph and (**b**) the trapezoidal piezoelectric bimorph.

The geometry of a piezoelectric bimorph is generally rectangular, but with this structure, it is difficult to provide the displacement and force needed by the alignment system. Furthermore, it has been reported that the trapezoidal piezoelectric bimorph has advantages in force and torque output [26]. In order to clarify the output difference between the two bimorphs, we established a rectangular piezoelectric bimorph model and a trapezoidal piezoelectric bimorph model. The two models were almost the same, except the trapezoid model had a wider baseline. Figure 2 shows the results of finite element simulations of the displacement of the actuators when a voltage of 100 V was applied.

From the results of the simulations, the terminal displacement of the rectangular bimorph was 85.2 μm, and that of the trapezoidal one was 86.9 μm, indicating that there was no significant difference in their output displacement.

**Figure 3:**
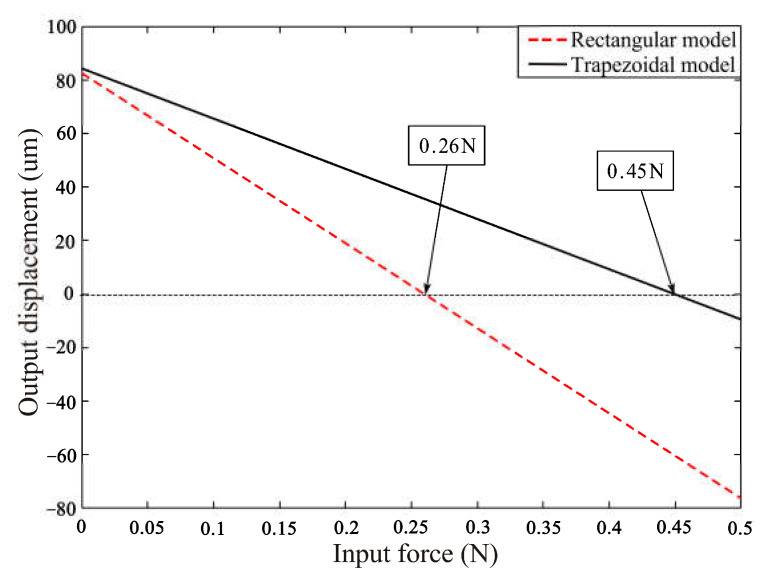
Relationship between output displacement and input force of the two piezo bimorphs.

Unlike measuring the terminal displacement, the output force cannot be calculated directly. As a pair of input and output forces are opposite but have the same value, the output force can be compared indirectly by the relationship between the output displacement and input force. To obtain the relationship, variable force is applied at the end of the bimorph, and its position will change correspondingly. The result is shown in Figure 3.

Based on the simulation results, the output displacement of the bimorphs changed linearly with the input force. The output capacity of the bimorphs can be reflected by the input force whose output displacement is 0. In this case, the maximum output force of the rectangular bimorph was 0.26 N, and that of the trapezoidal bimorph was 0.45 N. Compared with the rectangular bimorph, the trapezoidal one had the same output displacement but a larger output force, and was better for aligning the input fiber.

**Figure 4:**
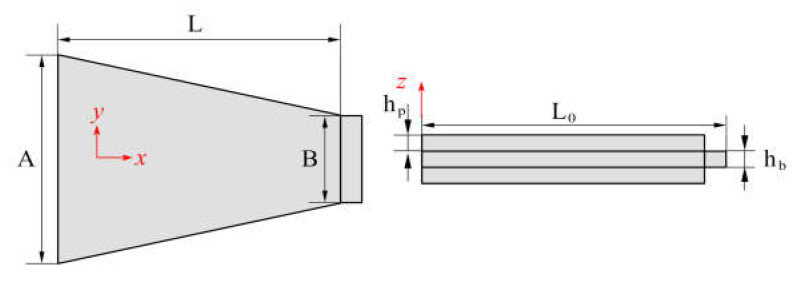
Structural parameters of the trapezoidal piezoelectric bimorph.

The structural parameters of the piezoelectric actuator are shown in Figure 4, where the width of the actuator baseline is *A*, the length of the piezoelectric layer is *L*, the thickness of the piezoelectric layer is *h_p_*, and the thickness of the metal layer is *h_b_*. The length of the middle layer is longer than other layers on the topline and is for installing a device to push the input fiber and avoid applying direct external forces on the piezoelectric material. According to the finite element simulation analysis and the technical requirements of the alignment system, the structural parameters of the piezoelectric twin chip were preliminarily determined, as shown in Table 1.

## 3. Mathematical Model and Verification

### 3.1. Subsection

The piezoelectric actuator here is a five-layer composite structure. The material of the middle layer is beryllium bronze, with PZT on both sides, and they are bonded together by epoxy resin. The output performance of the analysis below is based on the theory of laminates and material mechanics.

To simplify the calculation, we made the following assumptions [27,28]:

- The PZT-5H piezoelectric layer is completely attached to the middle layer so that there is no shear strain between the layers;
- The thickness of the adhesive layers can be ignored because they are much thinner than other layers;
- The width of the piezoelectric drive is much greater than its thickness;
- Piezoelectric materials are isotropic;
- The length of the neutral axis will not be changed, hence the strain on each layer is linear along the thickness direction;
- There is no axial load, only the load applied to the end of the actuator.

The first type of piezoelectric equation can be simplified as

(1) $$\left\{\begin{array}{l} \varepsilon_{1}=s_{11}^{E}\delta_{1}+d_{31}E_{3}\\ D_{3}=d_{31}\delta_{1}+c_{3}^{T}E_{3} \end{array}\right.,$$

where the lower corner indices 1, 2 and 3 represent the directions of X, Y, and Z axes, respectively, *ε*_1_ and *δ*_1_ are strain and stress in the *X*-axis direction, respectively, *E*_3_ and *D*_3_ are electric field strength and electric displacement in the *Z*-axis direction, respectively, *d*_31_ is coefficient of piezoelectric strain, *c^T^*_3_ is dielectric constant when the external stress is constant or 0, and $s_{11}^{E}$ is the elastic compliance coefficient when the electric field strength is constant or 0.

The strain of the *i*th layer in a laminated plate can be expressed as

(2) $$\varepsilon_{i}=s_{i}^{E}\delta_{i}+d_{31}E_{i}p,$$

where $s_{i}^{E}$ is the elastic modulus in the length direction, *δ_i_* is the stress tensor, *E_i_* is the electric field strength applied at the layer *i*, *p* is 1 if the directions of polarization and the electric field are the same, while *p* is −1 if they are opposite. Parameters in Equation (2) are of the *i*th layer.

If the distance from the fixed end to the measured point of the actuator is ***x***, the displacement of this point is

(3) $$v(x)=\frac{6d_{31}E_{p}V(h_{b}+h_{p})x^{2}}{6E_{p}h_{b}^{2}h_{p}+8E_{p}h_{p}^{3}+12E_{p}h_{b}h_{p}^{2}+E_{b}h_{b}^{3}},$$

**Figure 5:**
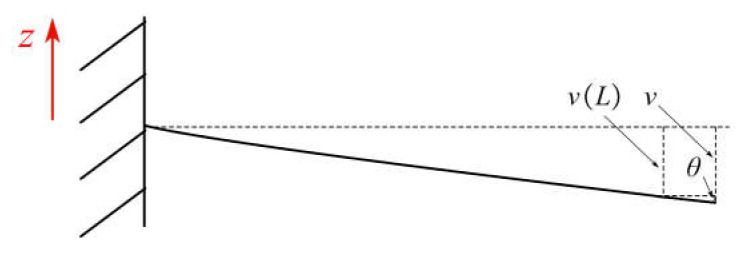
Output displacement of the actuator.

The extension part of the actuator middle layer is not subjected to external force. It can be considered to have no deformation when the bimorph is bending. Setting the deflection angle as *θ*, it approximately equals tan *θ* due to the small value of *θ*. The diagram of *θ* is shown in Figure 5. 

Substituting *x* = *L* into Equation (3), then *θ* is equal to

(4) $$\theta =\frac{12d_{31}E_{p}V(h_{b}+h_{p})L}{6E_{p}h_{b}^{2}h_{p}+8E_{p}h_{p}^{3}+12E_{p}h_{b}h_{p}^{2}+E_{b}h_{b}^{3}},$$

Let the total length of the actuator be *L*_0_, then the output displacement is equal to

(5) $$\begin{array}{l} v=v(L)+(L_{0}-L)\theta \\ \;\;\;=\frac{6d_{31}E_{p}V(h_{b}+h_{p})(2L_{0}-L)L}{6E_{p}h_{b}^{2}h_{p}+8E_{p}h_{p}^{3}+12E_{p}h_{b}h_{p}^{2}+E_{b}h_{b}^{3}} \end{array},$$

In the following parts, the mathematical model can be analyzed by MATLAB.

### 3.2. Verification with Mathematical and Finite Element Models

**Figure 6:**
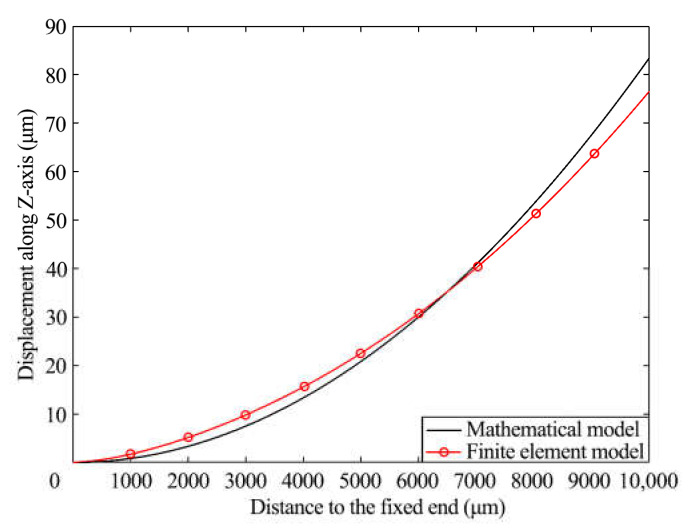
Comparison of numerical simulation and finite element simulation.

COMSOL is a multi-physics simulation software with the function of finite element analysis. Using MATLAB and COMSOL, we simulated the mathematical model and the finite element model and obtained the deformation curves of the two models along the *X*-axis, respectively. In Figure 6, the two simulation results are imported into one coordinate system. By comparison, the two lines changed similarly, which preliminarily proved the reliability of the two models. The maximum change in model position was located at *x* = 10 mm, where the displacement was about 85 μm beyond the basic requirement of 50 μm.

## 4. Output Displacement Analysis

Following is an analysis of the variation in the output displacement with increasing structural parameters including the width of the trapezoidal baseline (*A*), length and thickness of the piezoelectric layer (*L* and *h_p_*, respectively), and ratio of the metal layer thickness to the piezoelectric layer thickness (*r_h_*). The structural parameters of the actuator can be adjusted according to the analysis results.

### 4.1. Output Displacement Influenced by Width of Trapezoidal Baseline

**Figure 7:**
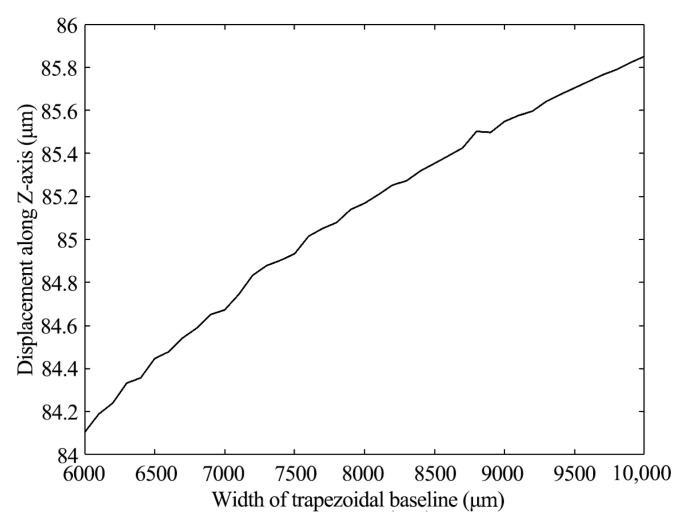
Output displacement and width of the piezoelectric layer.

From the mathematical functions, the value of the displacement is independent of the width of the trapezoidal baseline (*A*). The relationship between the two variables can only be simulated based on the finite element model. Before sweeping and analyzing the finite element model, *A* was set to move from 6000 μm to 10,000 μm, keeping other parameters unchanged. As shown in Figure 7, the change in output displacement was less than 1.5 μm, which was much smaller than the length of the actuator. Therefore, there was no obvious relation between *A* and the output displacement.

### 4.2. Output Displacement Influenced by Length of Piezoelectric Layer

**Figure 8:**
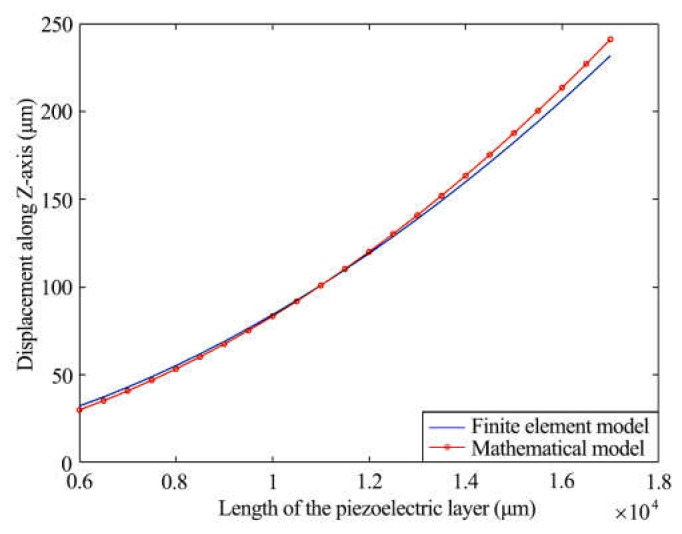
Output displacement and length of the piezoelectric layer.

The piezoelectric layers are used as the key parts for actuation. Considering the working environment and rigidity of the actuator, the length of the piezoelectric layers (*L*) was set to increase from 6000 μm to 18,000 μm, while other parameters were kept constant. After mathematical and finite element simulations, the results showed two similar curves representing the relationship between *L* and the output displacement, as shown in Figure 8. The output displacement reached 50 μm driven by 8000 μm long piezoelectric layers, and it increased to 200 μm if *L* was 16,000 μm. The relationship indicates that *L* has a great influence on the output displacement.

### 4.3. Output Displacement Influenced by Thickness of Piezoelectric Layer

**Figure 9:**
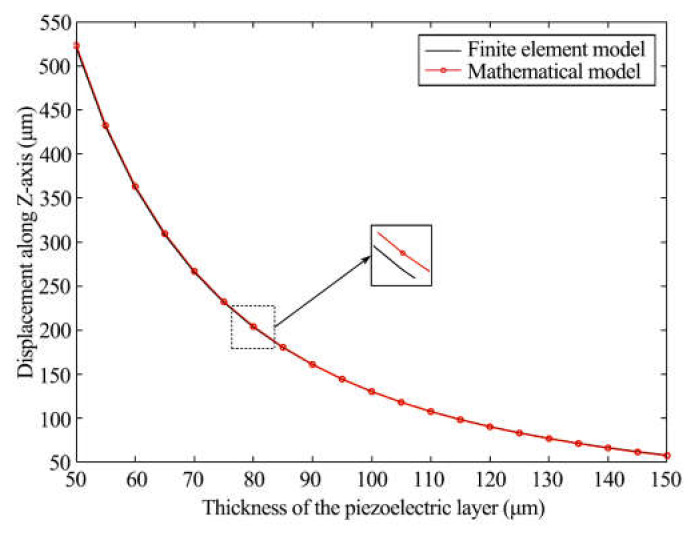
Output displacement and thickness of the trapezoidal baseline.

We studied the relationship between the output displacement and thickness of the piezoelectric layer (*h_p_*), with *h_p_* changing from 50 μm to 150 μm due to the stability and deformability of the actuator and impact of the material processing, and keeping the other parameters the same. Figure 9 shows the results of the two model simulations. The two curves are almost overlapping, and their values for the output displacement trend down with increasing *h_p_*. This demonstrates that a thinner bimorph produces greater output displacement. *h_p_* has a noticeable impact on the output displacement.

### 4.4. Output Displacement Influenced by Thickness Ratio of Metal Layer to Piezoelectric Layer

**Figure 10:**
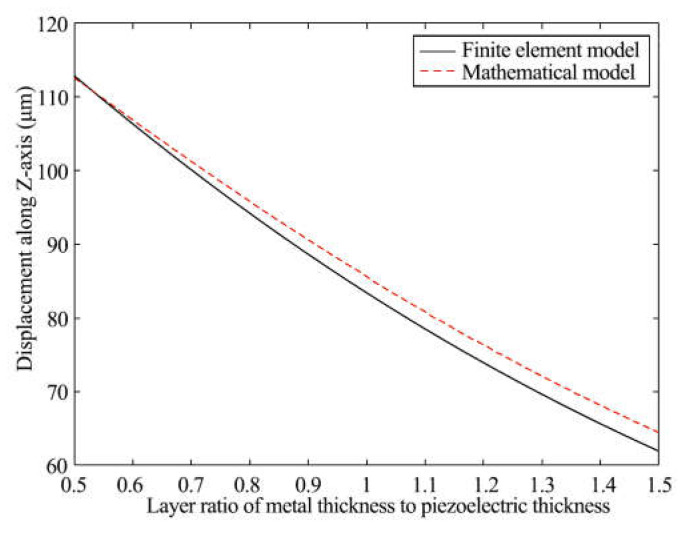
Output displacement and ratio of each layer thickness.

The thickness range of the metal layer is wider than that of the piezoelectric layer because of different manufacturing technologies. With the simulations and characteristics of the actuator above, the thickness of the piezoelectric layers was maintained at 125 μm. Constrained by the manufacturing process for beryllium bronze, the ratio of metal matrix layer thickness to piezoelectric layer thickness (*r_h_*) rose from 0.5 to 1.5. The results of the simulations are shown in Figure 10. The output displacement of the driver dropped smoothly as *r_h_* increased. Additionally, the curves of the two models were similar when *r_h_* was 0.5. As *r_h_* increased, the gap between the two curves grew.

### 4.5. Adjustment of Structural Parameters

Considering the analysis results above, the structural parameters for application could be ultimately determined for the following experiments. Unfortunately, the minimum thickness of the piezoelectric layer that could be found was 150 μm, which would inevitably decrease the output displacement. To make sure that the output displacement increase was higher than 50 μm, the length of the piezoelectric layer ***L*** was increased to 14,000 μm. The other parameters were kept unchanged, and details are in Table 2

## 5. Hysteresis Model and Parameter Identification

### 5.1. Hysteresis Loop Measurement

**Figure 11:**
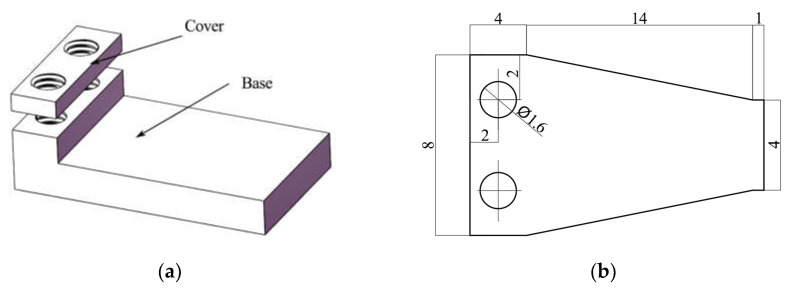
The prototype and its fixture. (**a**) The fixture of the actuator; (**b**) The dimensions of the metal layer.

A trapezoidal piezoelectric micro actuator and its adapted L-fixture were prepared for further experiments. The fixture consisted of a cover and a base made of acrylic or plastic. The structure of the L-fixture is shown in Figure 11a. The cover was provided to evenly clamp the actuator and wires with bolts, thus avoiding short circuits caused by direct connection of the wires and bolts. Compared with the structure of the piezoelectric actuator simulated above, there was an extra part at the trapezoidal baseline end of the metal layer. This part had two holes for passing the bolts and was clamped between the cover and the base to fix the actuator. Its structural parameters are shown in Figure 11b.

**Figure 12:**
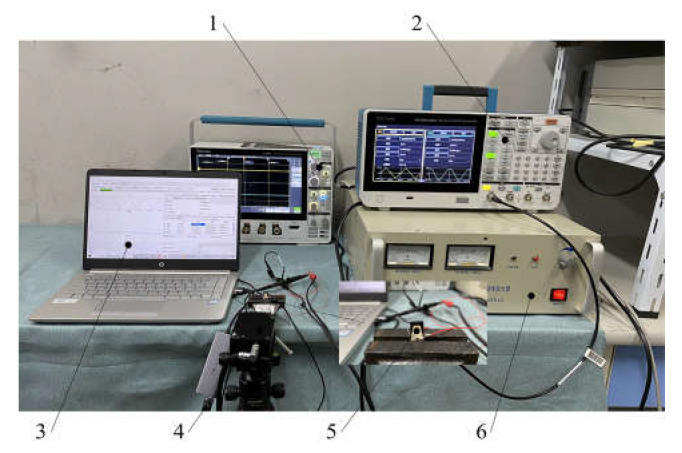
Picture of experimental platform. 1. Oscillograph; 2. signal generator; 3. PC; 4. laser doppler vibrometer; 5. prototype of the actuator; 6. power amplifier.

To test the hysteresis loop of the actuator, we set up an experimental platform that was composed of signal generator, power amplifier, oscilloscope, Doppler laser vibrometer and driver prototype, as shown in Figure 12. The laser Doppler vibrometer was a model KV-HB1525S. Integrated with a data acquisition module, this device collected data of the actuator and transmitted it to a computer, realizing real-time display and data preservation.

To generate actuating voltage, the signal generator produced a triangular wave with amplitude of 5 V, bias of 2.5 V and frequency of 0.1 Hz. Thanks to the power amplifier, the amplitude and the bias were amplified to 72 V and 36 V, respectively. The output displacement measured by the laser vibrometer varied with voltage, but its curve over time was not a regular triangular waveform, which means hysteresis existed on the actuator.

**Figure 13:**
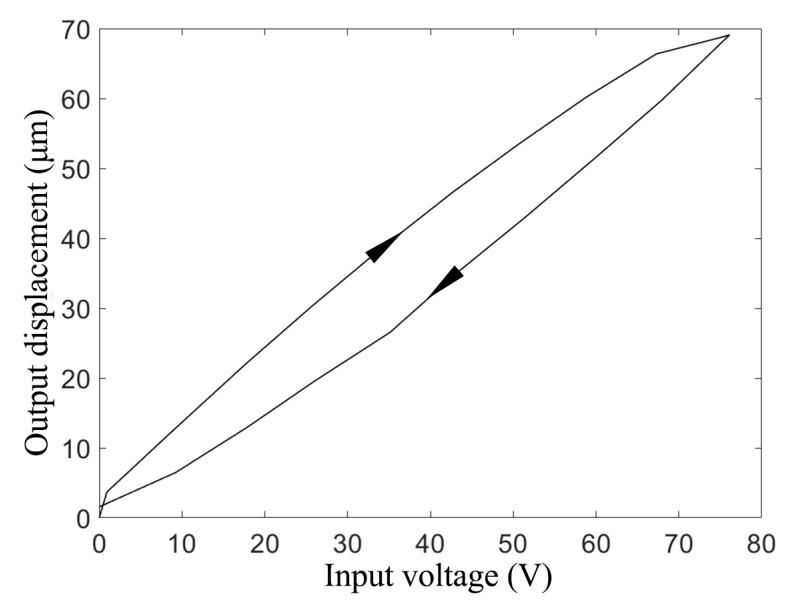
Hysteresis loop of the piezo actuator prototype.

For the purpose of examining the hysteresis loop, the variation in the output displacement should be tested after powering on the actuator for some time to avoid influence by the initial state of the actuator. The results are shown in Figure 13. The maximum output displacement was approximately 70 μm, which was smaller than that obtained by the simulation (89 μm). This was mainly attributable to assembly error caused by manual operation, and the welding spot may have affected the output as well.

### 5.2. Prandtl–Ishlinskii Model

**Figure 14:**
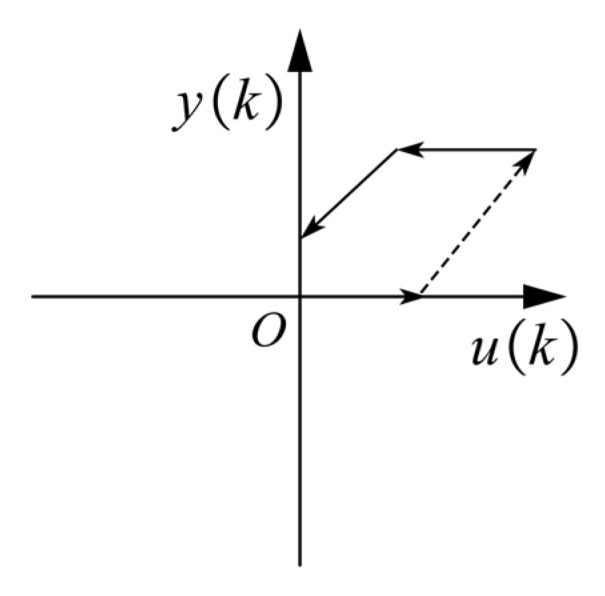
A part of the complete play operator.

The PI hysteresis model is an integral hysteresis model and a superposition of elementary play or stop operators. In following experiments, we set the input voltage *u*(*k*) as the input of the hysteresis operator, which was always positive, while its output was the output displacement *y*(*k*). Therefore, we only used a part of the complete play operator, as shown in Figure 14.

In Figure 14, the dashed line represents the operator output when the driving voltage *u*(*k*) rises, and the solid line corresponds to the operator output of the falling driving voltage. Backlash hysteresis operator function expression is as follows,

(6) $$y=\left\{\begin{array}{l} w\cdot (x-r),\;x-\frac{y}{w}=r\\ c,\;\;\;\;-r<x-\frac{y}{w}<r\\ w\cdot (x+r),\;x-\frac{y}{w}=r \end{array}\right.,$$

where *x* and *y* represent the Backlash input and output, respectively, *w* is the weight coefficient corresponding to a hysteresis operator, and ***r*** is the selected threshold input.

Then, the threshold is divided according to the number of Backlash operators, and the Backlash operators are multiplied with their corresponding weight coefficients, respectively. The mathematical expression of the PI model [29] is

(7) $$Y(k)=\sum_{i=1}^{n}w_{i}\cdot y_{i}(k)=\sum_{i=1}^{n}w_{i}\cdot \max\left\{u(k)-r_{i},\min\left[u(k)+r_{i},y_{i}(k-1)\right]\right\},$$

where *Y*(*k*) represents the model output with the *k*th input, *n* is the number of operators, *w_i_* is the weight coefficient of the *i*th operator, and *r_i_* is the threshold of the *i*th operator.

### 5.3. Parameter Identification Based on Particle Swarm Optimization

The parameter identification method can be considered as determining the threshold and initial value of the PI model and identifying the weight coefficient of each threshold value. The division of threshold values can be expressed as

(8) $$r_{i}=\frac{i}{n}\max\left|u(k)\right|,i=0,1,\ldots n-1,r_{\max}<u_{\max},$$

PI model parameters can be identified based on the initial loading curve. Initial loading curve refers to the output displacement curve of an actuator while an initial loading voltage changes from zero to the maximum value. The curve is a piecewise linear function influenced by the threshold *r*, which is

(9) $$\varphi (r)=\sum_{j=0}^{i}w_{j}(r-r_{j});\;\;r_{i}\leq r<r_{i+1},\;i=0,1,\ldots ,n-1,$$

The slope of the curve at each threshold is the sum of weight coefficients before the threshold, which is

(10) $$\frac{d}{dr}\varphi (r)=\sum_{j=0}^{i}w_{j},$$

For efficient identification, particle swarm optimization is used due to its simple structure and fast calculation speed. The specific process is as follows:

1. Initialize the velocity of the particle.
  (11) $$v_{i}^{1}=v_{\min}+a(v_{\max}-v_{\min})\;\;\;\;\;\;\;\;\;\;\;\;\;\;\;\;\;\;\;\;\;i=1,2,\ldots n$$
2. Where $v_{i}^{1}$ represents the *i*th particle in the primary population, and *v*_max_ and *v*_min_ are the minimal and maximal speed set in the particle swarm optimalization algorithm, respectively.
3. To calculate the fitness of the particle, the fitness function is
  (12) $$J(t)=\sqrt{\frac{\sum_{i=1}^{N}{\left[x(t)-y(t)\right]}^{2}}{n}},$$
4. where *x*(*t*) are displacements from measurement and simulation model, respectively, when the time goes to *t*.
5. Initialize and calculate iteratively optimal positions of individual and global optimization.
6. Update the position and velocity of particles and generate new populations. To ensure that the updated speed and position are in the definition domain, *v*(*v* > *v*_max_) = *v*_max_ is generally an efficient method for measuring the maximal speed boundary. And, the idea is the same for measurements of minimal speed boundary and position boundaries, as follows:
  (13) $$v_{i}^{t+1}=v_{i}^{t}+c_{1}a_{1}(pb_{i}-x_{i}^{t})+c_{2}a_{2}(gb-x_{i}^{t}),$$
  (14) $$x_{i}^{t+1}=x_{i}^{t}+v_{i}^{t+1},$$
7. where ***c*_1_** and ***c*_2_** are vectors composed of self and group learning factors, respectively. *a*_1_ and *a*_2_ are vectors composed of random numbers that are in the range of [0, 1]. $x_{i}^{t}$ represents the position vector of the *i*th particle at the *t*th evolution.
8. Calculate the fitness value of each particle and compare it with the previously recorded individual optimal and global optimal positions. If the calculated fitness value is higher, it will be recorded as the new individual optimal or even global optimal position.
9. Set the maximal evolutionary generation as 150 and check whether the evolutionary number meets the ending condition. If the number reaches the setting, the algorithm can be ended, otherwise, turn back to step 4.

**Figure 15:**
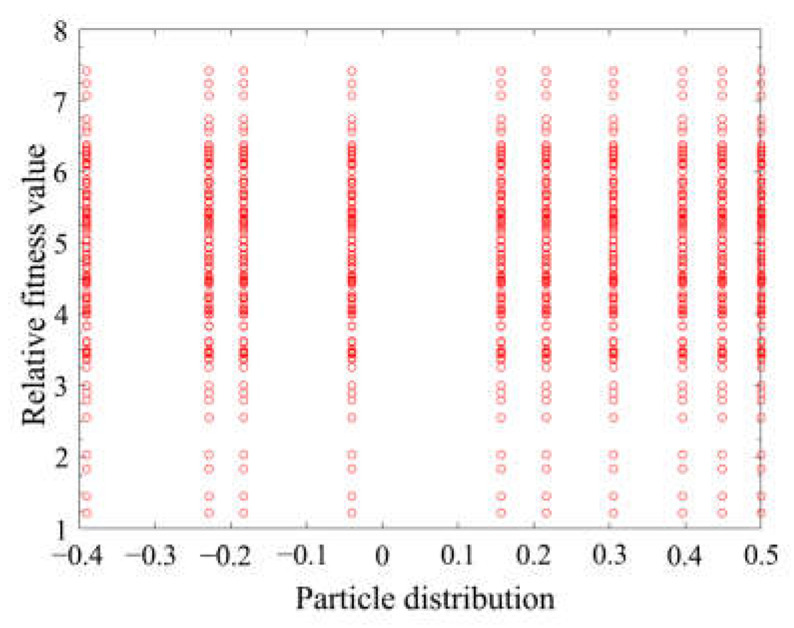
Particle distribution and fitness value of the 150th generation.

Due to the fast speed of particle swarm optimization, there is no need to normalize the data. Instead, the original data are used directly in parameter identification. In an identification process, particle positions and fitness values are randomly disordered at first, and they gradually change into regular distribution at last. The operation results for the 150th generation are shown in Figure 15. 

After the operation, it was found that the fitness value of 1.5432 converged at the 65th generation. Through identification, the parameters were as shown in Table 3.

**Figure 16:**
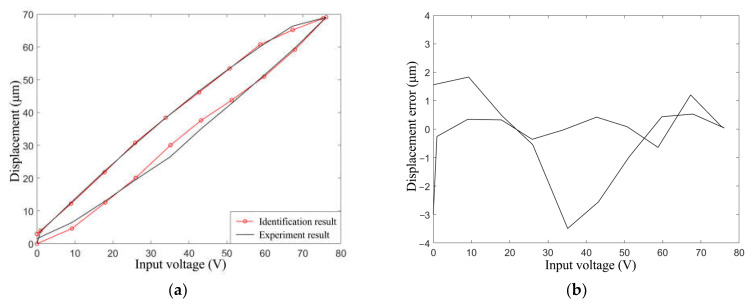
Comparing the fitted hysteresis curve and experimental hysteresis curve. (**a**) The two curves; (**b**) The error of fitting hysteresis curve and experimental hysteresis curve.

Substitute the parameters in Table 3 into the PI model so that the simulated hysteresis curve is obtained. The comparison of simulation and experimental hysteresis curves is shown in Figure 16a. It can be seen that the PI model with particle swarm optimization fits the experimental data well, which lays a foundation for reducing the hysteresis effect. The difference between the two curves is shown in the error diagram in Figure 16b. When the voltage is 30~50 V, the fitting error is large and the maximum value of the error is 3.5 μm. However, the model fits more accurately with other input voltages.

## 6. Control of the Piezo Actuator

There was an obvious nonlinear relationship between the output displacement and input voltage of the piezoelectric actuator. Based on the hysteresis model, we decided to examine the control effect of feedforward control and PID control. Finally, the two control methods were combined to improve output accuracy and reduce response time and overshoot.

### 6.1. Feedforward Control

The most common method of controlling the hysteresis of piezoelectric actuators is feedforward control. It reduces the impact of hysteresis using an inverse model of hysteresis and its compensation controller [30].

#### 6.1.1. Inverse Hysteresis Model

The matrix multiplication of a hysteresis model and its inverse model is an identity matrix *E*, which is *H*(*k*)*H*^−1^(*k*) = *E*. Then, the inverse PI model can be obtained as

(15) $$H^{-1}(k)=\sum_{i=1}^{n}{w^{\prime }}_{i}\cdot \max\left\{y(k)-{r^{\prime }}_{i},\min\left[y(k)+{r^{\prime }}_{i},u_{i}(k-1)\right]\right\},$$

where ${r^{\prime }}_{i}$ and ${w^{\prime }}_{i}$ are the thresholds and the weight coefficients of each operator in the inverse model, respectively, and can be expressed as

(16) $${w^{\prime }}_{1}=\frac{1}{w_{1}},{w^{\prime }}_{i}=\frac{-w_{i}}{\left[\left(\sum_{j=1}^{i}w_{j}\right)\left(\sum_{j=1}^{i-1}w_{j}\right)\right]}\;i=2,3,\cdots ,n,$$

(17) $${r^{\prime }}_{i}=\sum_{j=1}^{i}w_{j}(r_{i}-r_{j})\;i=1,2,\cdots ,n,$$

The initial value of the inverse model operator is

(18) $$u_{i}(0)=\sum_{j=1}^{i-1}w_{j}y_{i}(0)+\sum_{j=i}^{n}w_{j}y_{j}(0)\;i=2,3,\cdots ,n,$$

According to Table 3 and Equations (16)–(18), the model parameters of the PI hysteresis inverse model are as shown in Table 4.

#### 6.1.2. Feedforward Control Effect

**Figure 17:**
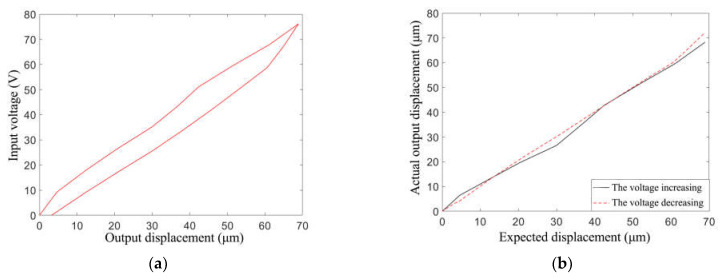
Results of feedforward control. (**a**) Inverse hysteresis model; (**b**) feedforward control effect.

To verify the feasibility of the designed controller, a control simulation analysis was carried out in MATLAB based on the models above. The inverse hysteresis model and the results of feedforward control are shown in Figure 17.

The output results in Figure 17b were studied separately depending on whether the input voltage rose or fell. Comparing the output of the actuator model and the expected output value, it was found that the relationship between them was roughly linear, while bias existed. Moreover, due to the lack of feedback, it was hard to correct bias with the feedforward controller. Therefore, other control methods should be studied for improvements in accuracy.

### 6.2. Fuzzy PID Control

In PID control, the output bias is looped back into the input signal, which provides an efficient solution for control systems that require feedback terms.

#### 6.2.1. Dynamic Model of the Piezo Actuator

The dynamic model of a piezoelectric bimorph actuator can be simplified as a mass-spring-damping system in the vertical position [31], and the mass (*m*), the damper (*c*) and the spring (*k*) represent equivalent mass, damping, and rigidity of the actuator’s piezo element, respectively. *m* is supported by *k* and *c*, which are connected in parallel. Additionally, *m* is pushed by the output force *f*(*t*), as the direction of *f*(*t*) and *y*(*t*) is straight up. The kinematic differential equation of the driver in the vertical direction is

(19) $$m\frac{d^{2}}{dt^{2}}y(t)=f(t)-ky(t)-c\frac{d}{dt}y(t),$$

In fact, the equivalent stiffness of the actuator *k_a_* is much larger than *k*. If the hysteresis effect is ignored, the transfer function can be simplified and expressed as follows:

(20) $$G(s)=\frac{\alpha k_{a}/m}{s^{2}+2w_{n}\xi s+w_{n}^{2}},$$

where α is the ratio of output displacement to drive voltage without considering the hysteresis, $w_{n}=\sqrt{k_{a}/m}$ represents the resonant frequency of the actuator in the *Z*-axis direction, and *ξ* = *c*/*2mw_n_* is the damping ratio of the actuator in the *Z*-axis direction.

#### 6.2.2. Fuzzy PID Control Effect

**Figure 18:**
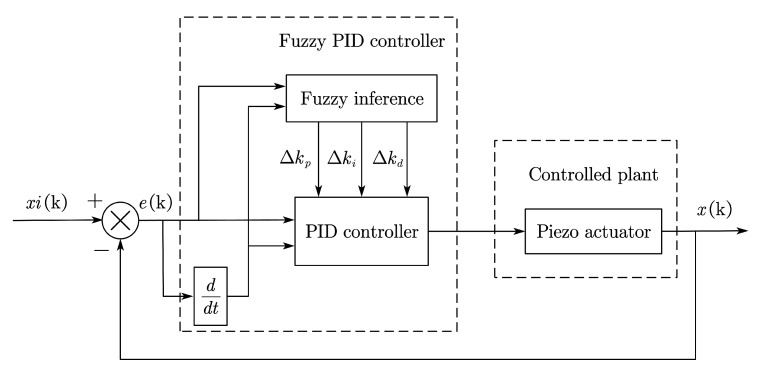
The principle of fuzzy PID control.

The classical PID control is widely used and has proportional, integral, and derivative terms. Different terms can be used individually or in combination. Nevertheless, the parameters of the three terms cannot be changed after they are determined, which has an adverse effect on controlling nonlinear systems. Therefore, combining classical PID with fuzzy control, the fuzzy PID can adjust parameters timely according to the real-time response of the operating system [16]. The control principle is shown in Figure 18.

The fuzzy control can be divided into four steps: fuzzifying the crisp input information, establishing the fuzzy rule charts, processing fuzzified information using the fuzzy rule charts, and converting the fuzzy control signal into crisp output directly usable for the actuator.

Before fuzzification, the domain of input and output values should be determined firstly. The inputs of the fuzzy controller include the deviation signal (E) and the variation rate of deviation signal (EC) of the output displacement, while the outputs are the coefficient change of proportional term (*k_p_*), integral term (*k_i_*), and derivative term (*k_d_*). Considering the variation in the input signal, the discourse domain of E is defined as [−12, 12], and that of EC is defined as [−20, 20]. According to the variation of the output signal, the discourse domains of *k_p_*, *k_i_*, and *k_d_* are defined as [−6, 6], [−4, 4] and [−3, 3], respectively. Additionally, the fuzzy subset of a domain is presented as {FD, FZ, FX, Z, ZX, ZZ, ZD}, which is ordered from the smallest to the largest and defined as 0 at level Z.

**Figure 19:**
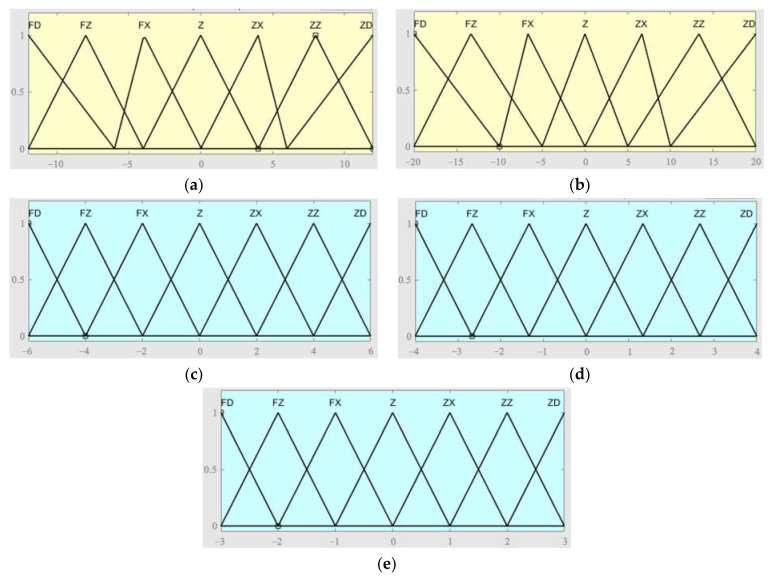
Membership functions of each parameter. (**a**) Membership function of E. (**b**) Membership function of EC. (**c**) Membership function of *k_p_*. (**d**) Membership function of *k_i_*. (**e**) Membership function of *k_d_*.

The fuzzification process for the inputs and the outputs cannot proceed well without appropriate membership functions. We decided to use triangular membership functions according to the changing law of variables and the expected control effect, as shown in Figure 19. Based on the three outputs of the fuzzy PID controller and the fuzzy subset, the corresponding fuzzy rule inference tables were developed, as shown in Table 5, Table 6 and Table 7.

Because the fuzzy results from the fuzzy interference are not capable of controlling the system, the fuzzy control signals should be converted into crisp formulations. The method of center-of-gravity (COG) is commonly used for defuzzification because the output will change smoothly if the input changes smoothly as well. The defuzzification result is the gravity center of a region enclosed by the membership function and the *X*-axis. The defuzzification can be expressed as

(21) $$x^{*}=\frac{\int x\mu_{c}(x)dx}{\int \mu_{c}(x)dx},$$

The precise control quantities can be obtained by defuzzification conversion, which realizes the self-tuning of the parameters in the PID. The control quantities are expressed as

(22) $$\left\{\begin{array}{c} k_{p}=k_{p0}+\Delta k_{p}\\ k_{i}=k_{i0}+\Delta k_{i}\\ k_{d}=k_{d0}+\Delta k_{d} \end{array}\right.,$$

where *k_p_*_0_, *k_i_*_0,_ and *k_d_*_0_ are the initial values of proportional, integral, and differential terms, respectively, while ∆*k_p_*, ∆*k_i,_* and ∆*k_d_* are the changes in the above three parameters, respectively.

The model of the fuzzy controller designed above was built in Simulink. Subsequently, several experiments were carried out to determine that the scale factors of *k_p_*, *k_i,_* and *k_d_* were 0.8, 0.8, and 0.6, respectively. The initial parameters of the PID controller were the same as the parameters selected earlier.

**Figure 20:**
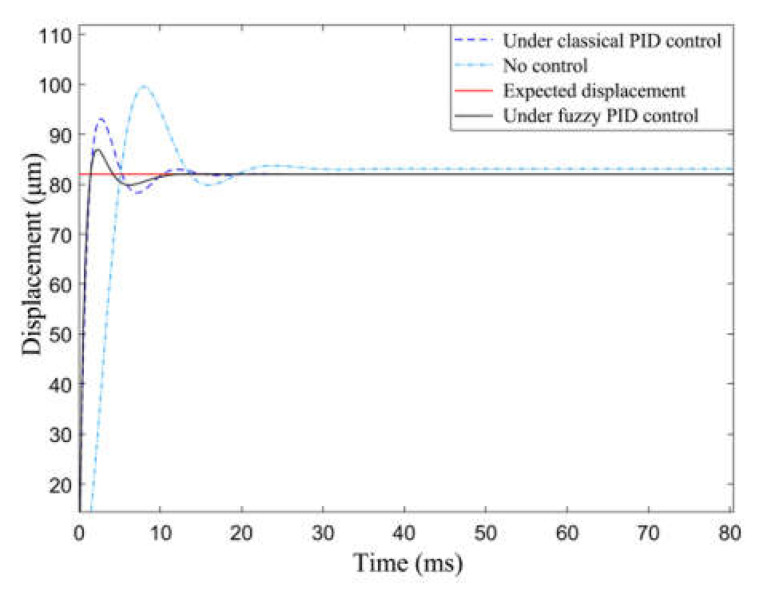
Fuzzy PID control simulation results and results under other conditions.

To clarify the characteristics of the fuzzy PID algorithm, the output displacement of the actuator with no control, under classical PID control, and under fuzzy PID control were compared with the objective output displacement. When the step signal of the objective output displacement was 82 μm, the corresponding simulation results were as shown in Figure 20. The figure reveals that the fuzzy PID controller can reduce fluctuations in the control system whose response time is 15 ms and overshoot is 5% compared with the classical PID control. The controlling effect of the fuzzy PID control is better than that of the classical PID control.

### 6.3. A Composite Control and Displacement Tracking Simulation

**Figure 21:**
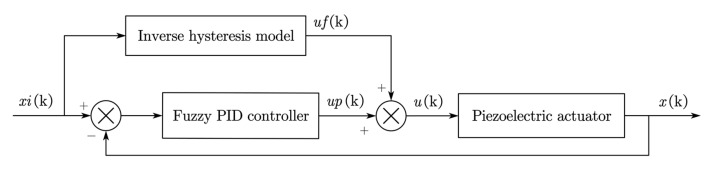
The principle of the composite control.

Since the PID control in this paper did not take the piezoelectric hysteresis effect into consideration, we added a composite control to the piezoelectric micro actuator in order to investigate further. Firstly, the objective output displacement was substituted into the inverse hysteresis model to obtain the corresponding driving voltage. Then, we added the output voltages from the inverse model and the fuzzy PID controller, and their sum was exerted to motivate the actuator. The principle of the composite control is shown in Figure 21. 

The transfer function of the composite control system is

(23) $$\begin{array}{c} G(k)=\frac{x(k)}{u(k)}\\ =\frac{\sum_{i=1}^{n}w_{i}\cdot \max\left\{u(k)-r_{i},\min\left[u(k)+r_{i},xi(k-1)\right]\right\}}{\sum_{i=1}^{n}w_{i}{}^{\prime }\cdot \max\left\{u(k)-{r^{\prime }}_{i},\min\left[u(k)+{r^{\prime }}_{i},xi(k-1)\right]\right\}+k_{p}e(k)+k_{i}\sum_{i=0}^{k}e(i)+k_{d}[e(k)-e(k-1)]} \end{array}$$

**Figure 22:**
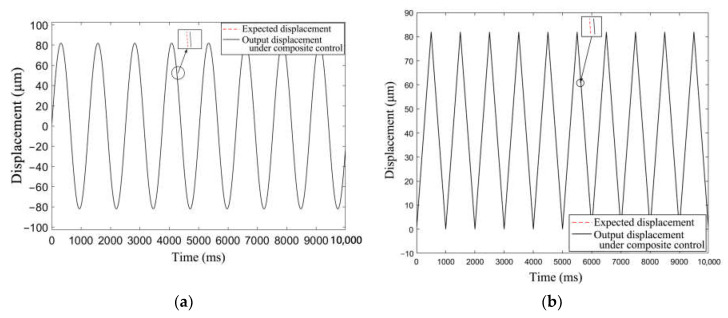
The response curves of the output displacement under the composite control. (**a**) The input signal is a sinusoidal signal. (**b**) The input signal is a triangular signal.

To verify the controller’s ability of tracking signals with periodic changes, a simulation analysis was carried out, ignoring external interference signals. During the analysis process, the input signal was an unbiased sine wave signal with a frequency of 0.75 Hz and amplitude of 82 μm, and the total simulation time was 10,000 ms. Then, the input signal was changed into a triangular signal with a frequency of 1 Hz, an amplitude of 82 μm, and a bias of 41 μm, while the simulation time remained unchanged. The simulation results of the two situations are shown in Figure 22.

It was observed that the system could track the periodically varying input signal, and the fitting degree of the target displacement and the tracking displacement under the compound control was high. After error analysis, the tracking error of the sinusoidal signal was less than 0.2 μm, while that of the triangular wave signal was less than 0.08 μm. We found that the displacement tracking error of the controller was small, so the control precision of the system met the design requirements.

## 7. Conclusions

In this paper, a trapezoidal piezoelectric bimorph actuator for optical fiber alignment was proposed. Further, its characteristics were analyzed, and the method of controlling the actuator was studied as well. The output displacement of the actuator was higher than 65 μm, and the minimum response time was reduced to 15 ms. Also, the periodic signal tracking accuracy met the design requirements. The design provides an actuator for directly aligning optical fibers. Moreover, the analysis of the actuator characteristics and control methods can be helpful for actuator design in practical applications. However, some research points should be addressed in the next study stage. For example, output force should be studied, and the composite control should be evaluated experimentally to prove its feasibility.

## Figures and Tables

**Table 1:** Initial structural parameters of the piezoelectric actuator.

Parameter	*A*	*B*	*L*	*L*_0_	*h_p_*	*h_b_*
Value (μm)	8000	4000	10,000	10,500	125	125

**Table 2:** Final structural parameters of the piezoelectric actuator.

Parameter	*A*	*B*	*L*	*L*_0_	*h_p_*	*h_b_*
Value (μm)	8000	4000	14,000	10,500	150	125

**Table 3:** The values of the parameters after identification.

	Threshold Value	Weight Coefficient		Threshold Value	Weight Coefficient
1	0	0.4437	6	40	−0.4379
2	8	0.4327	7	48	0.4215
3	16	0.0600	8	56	0.2314
4	24	0.1557	9	64	−0.1121
5	32	−0.0923	10	72	−0.3278

**Table 4:** The values of the parameters obtained through calculation.

	Threshold Value	Weight Coefficient		Threshold Value	Weight Coefficient
1	0	2.2536	6	40	0.0225
2	8	−1.1126	7	48	−0.0158
3	16	−0.0251	8	56	−0.0059
4	24	−0.0238	9	64	0.0020
5	32	0.0071	10	72	0.0047

**Table 5:** Fuzzy rule inference table of *k_p_*.

*k_p_*		EC						
		FD	FZ	FX	Z	ZX	ZZ	ZD
E	FD	ZD	ZD	ZZ	ZZ	ZX	Z	Z
	FZ	ZD	ZD	ZZ	ZX	ZX	Z	FX
	FX	ZZ	ZZ	ZZ	ZX	Z	FX	FX
	Z	ZZ	ZZ	ZX	Z	FX	FZ	FZ
	ZX	ZX	ZX	Z	FX	FX	FZ	FZ
	ZZ	ZX	Z	FX	FZ	FZ	FZ	FD
	ZD	Z	Z	FZ	FZ	FZ	FD	FD

**Table 6:** Fuzzy rule inference table of *k_i_*.

*k_i_*		EC						
		FD	FZ	FX	Z	ZX	ZZ	ZD
E	FD	FD	FD	FZ	FZ	FX	Z	Z
	FZ	FD	FD	FZ	FX	FX	Z	Z
	FX	FD	FZ	FX	FX	Z	ZX	ZX
	Z	FZ	FZ	FX	Z	ZX	ZZ	ZZ
	ZX	FZ	FX	Z	ZX	ZX	ZZ	ZD
	ZZ	Z	Z	ZX	ZX	ZZ	ZD	ZD
	ZD	Z	Z	ZX	ZZ	ZZ	ZD	ZD

**Table 7:** Fuzzy rule inference table of *k_d_*.

*k_d_*		EC						
		FD	FZ	FX	Z	ZX	ZZ	ZD
E	FD	ZX	FX	FD	FD	FD	FZ	ZX
	FZ	ZX	FX	FD	FZ	FZ	FX	Z
	FX	Z	FX	FZ	FZ	FX	FX	Z
	Z	Z	FX	FX	FX	FX	FX	Z
	ZX	Z	Z	Z	Z	Z	Z	Z
	ZZ	ZD	FX	ZX	ZX	ZX	ZX	ZD
	ZD	ZD	ZZ	ZZ	ZZ	ZX	ZX	ZD

## Data Availability

The data presented in this study are available on request from the corresponding author.
